# Heterologous Expression of a Thermostable α-Galactosidase from *Parageobacillus thermoglucosidasius* Isolated from the Lignocellulolytic Microbial Consortium TMC7

**DOI:** 10.4014/jmb.2201.01022

**Published:** 2022-05-16

**Authors:** Yi Wang, Chen Wang, Yonglun Chen, MingYu Cui, Qiong Wang, Peng Guo

**Affiliations:** 1 Institute of Agricultural Products Processing and Nuclear Agriculture Technology Research, Hubei Academy of Agricultural Sciences, Wuhan 430064, P.R. China; 2 College of Biology and Pharmacy, Three Gorges University, Yichang 443002, P.R. China

**Keywords:** α-Galactosidase, thermophilic enzyme, enzymatic properties, GH36, heterologous expression

## Abstract

α-Galactosidase is a debranching enzyme widely used in the food, feed, paper, and pharmaceuticals industries and plays an important role in hemicellulose degradation. Here, T26, an aerobic bacterial strain with thermostable α-galactosidase activity, was isolated from laboratory-preserved lignocellulolytic microbial consortium TMC7, and identified as *Parageobacillus thermoglucosidasius*. The α-galactosidase, called T26GAL and derived from the T26 culture supernatant, exhibited a maximum enzyme activity of 0.4976 IU/ml when cultured at 60°C and 180 rpm for 2 days. Bioinformatics analysis revealed that the α-galactosidase T26GAL belongs to the GH36 family. Subsequently, the pET-26 vector was used for the heterologous expression of the T26 α-galactosidase gene in *Escherichia coli* BL21 (DE3). The optimum pH for α-galactosidase T26GAL was determined to be 8.0, while the optimum temperature was 60°C. In addition, T26GAL demonstrated a remarkable thermostability with more than 93% enzyme activity, even at a high temperature of 90°C. Furthermore, Ca^2+^ and Mg^2+^ promoted the activity of T26GAL while Zn^2+^ and Cu^2+^ inhibited it. The substrate specificity studies revealed that T26GAL efficiently degraded raffinose, stachyose, and guar gum, but not locust bean gum. This study thus facilitated the discovery of an effective heat-resistant α-galactosidase with potent industrial application. Meanwhile, as part of our research on lignocellulose degradation by a microbial consortium, the present work provides an important basis for encouraging further investigation into this enzyme complex.

## Introduction

Lignocellulose has a complex structure comprising cellulose, hemicellulose, and lignin. The biological degradation of lignocellulose has received considerable attention because of its high efficiency and cost-effectiveness in addition to its low-carbon features [1]. Several studies have demonstrated that microbial consortia are particularly advantageous for the biotransformation of lignocellulose owing to the presence of a highly efficient synergistic multi-enzyme complex [2, 3]. In contrast to the traditional method of isolating strains and enzymes directly from the environment, exploring lignocellulose-degrading enzymes from the lignocellulose-degrading composite microbial systems is more efficient as it is more advantageous in the subsequent construction of lignocellulose-degrading enzyme complexes [4]. The microbial consortium TMC7, which is highly efficient in decomposing natural lignocellulose, was constructed previously in our laboratory. Metagenomics studies have revealed that TMC7 encoded abundant CAZymes (carbohydrate-active enzymes) associated with lignocellulose degradation [5]. Among these CAZymes, the degradation of hemicellulose is particularly dependent on the synergy of enzymes associated with the debranching and degradation of the hemicellulose backbone [5, 6].

α-Galactosidase (E.C. 3.2.1.22) is an exo-acting glycoside hydrolase that specifically catalyzes the breakage of the α-galactosyl unit on the non-reducing terminal of α-galactooligosaccharides and galactomannans [7, 8], thereby promoting the degradation of hemicellulose in lignocellulose. In addition, α-galactosidase has wide application in the food, feed, paper, and pharmaceutical industries [9]. Soybean products and feeds contain many oligosaccharides that monogastric animals cannot decompose. The fermentation of these oligosaccharides by microorganisms at the rear end of the intestine leads to the production of gases, which could cause gastrointestinal discomfort and affect the digestion of nutrients [10, 11]. Consequently, the addition of α-galactosidase in soybean products and feed can facilitate efficient degradation of the oligosaccharides, and reduce the adverse reaction associated with their consumption while improving nutritional assimilation. In the sugar industry, raffinose hinders the leaching of sucrose, whereas the addition of α-galactosidase has been shown to improve the leaching rate of sucrose [12]. In medicine, α-galactosidase has been documented to convert blood type B into type O and ease the supply shortage of certain blood types. Furthermore, α-galactosidase is reported to treat Fabry disease [13, 14], while a heat-resistant α-galactosidase could provide particular advantage. For instance, the pasteurization of soybean milk and other processes such as pelleting, puffing of feed, and sugar refining are accompanied by high temperature [15].

Accordingly, in this study, a strain with α-galactosidase activity and named T26, was isolated in-house from our own lignocellulolytic microbial consortium TMC7. The T26 strain was identified by 16S rDNA sequencing and the enzymatic properties of the α-galactosidase T26GAL were analyzed. The genomic DNA of T26 was then used as a template to obtain the *T26gal* gene by polymerase chain reaction (PCR). Amplification and bioinformatics methods were employed to analyze the structure of *T26gal*, which was subsequently cloned into pET26b (+) vector and heterologously expressed in *Escherichia coli* BL21 (DE). This study thus facilitated the identification of an effective heat-resistant α-galactosidase with promising potential for industrial application. Finally, the present work provides an important basis for research on the enzyme complexes associated with the lignocellulolytic microbial consortium.

## Materials and Methods

### Isolation of Strain T26

The microbial consortium TMC7 is a thermophilic lignocellulolytic microbial consortium generated in our previous study [16]. For the isolation of T26, the TMC7 was activated and inoculated in PCS medium (containing 10 g/l alkali-treated straw, 1 g/l peptone, 5 g/l NaCl, 2 g/l yeast powder, 2 g/l CaCO_3_, 0.35 g/l MgSO_4_·7H2O, and 1 g/l K_2_HPO_4_), followed by culturing in the dark under static conditions at 65°C for 7 days. Five milliliters of the culture samples were taken each day and pooled. A gradient dilution method was performed to dilute the mother liquor concentration to 10^-5^ and 10^-6^, and 100 μl of the diluted sample was spread onto LB plates (containing 10 g/l tryptone, 5 g/l yeast powder, 5 g/l NaCl, and 1.5% agar) and incubated upside down at 60°C for 7 days. Three replicates were performed for each dilution gradient. Subsequently, three replicates of streaking were performed for the isolated strains. The T26 strain used in the present study was one of the isolates and was preserved in 20%glycerol (v/v) at -80°C.

Single colonies of T26 were placed into the liquid LB medium and incubated at 60°C under 180 rpm for 4 days. Five milliliters of the culture samples were collected each day and the pH of the culture was determined using a pH meter (B-212; Horiba, Ltd., Japan). The protein content of the culture samples was determined by Bradford protein assay [17]. The biomass of the culture was determined by measuring the optical density (OD) at 600 nm.

### Genotypic Identification and Biochemical Characterization of T26 Isolate

Gram staining of freshly cultured T26 was performed and the results were determined microscopically. The total genomic DNA of T26 was extracted using a PureLink Genomic DNA Kit (Thermo Fisher Scientific, USA). The primers 27F (5’-AGAGTTTGATCCTGGCTCAG-3’) and 1492R (5’-TACGGCTACCTTGTTACGACTT-3’) were used for the 16S rDNA amplification using DreamTaq DNA Polymerase (Thermo Scientific). The products were ligated to the pTOPO-TA vector (Thermo Scientific), and the positive clones were selected and dispatched for sequencing to Tsingke Biotechnology Co., Ltd., China. The sequencing data were aligned using the NCBI BLAST (https://novopro.cn/blast/), and a phylogenetic tree was constructed using MEGA 7.0. The biochemical characteristics of the T26 strain were determined using API 50CHB strips (bioMérieux SA, France) according to the manufacturer's instructions [18]. After proper mixing, the T26 culture was transferred into API 50CHB ampules and incubated at 60°C for 24 h. The color change of the API 50CHB strip from red to yellow indicated positive results.

### Determination of T26GAL Enzyme Activity

The enzyme activity of T26-derived α-galactosidase was determined by a modified p-nitrophenyl-α-D-galactopyranoside (pNPGal) method [19]. Briefly, after incubation at 60°C for 5 min, 200 μl of 2 mg/mL pNPGal (purchased from MilliporeSigma., USA, dissolved in 0.01 M sodium phosphate buffer, pH 7.0) was mixed with 100 μl of the crude enzyme and then incubated at 60°C for 30 min. The reaction was stopped by adding 300 μL of 1 M sodium carbonate, and the absorbance was measured at 400 nm. One enzyme unit (U) was defined as a release of 1 μg of nitrophenol per minute.

### Effect of Temperature, pH, and Metal Ions on T26GAL Enzyme Activity

The T26GAL enzyme reaction was performed at 30, 40, 50, 60, 70, 80, and 90°C to determine the effect of temperature on the α-galactosidase activity. The highest enzyme activity was defined as 100% to calculate the relative change in the enzyme activity at differen*t* test temperatures.

To determine the effect of pH on T26GAL activity, pH tests were employed. In these tests, 0.01 M disodium hydrogen phosphate-citrate buffers at pH 3.0–5.0, 0.01 M phosphate buffers at pH 5.0–9.0, and 0.01 M sodium hydroxide-glycine buffers at pH 9.0–11.0 were used. The enzyme reaction was performed at pH ranging from 3.0 to 11.0 with relevant buffers to determine the effect of pH on enzyme activity. The highest enzyme activity was defined as 100% to calculate the relative enzyme activity.

The effect of metal ions including K^+^, Na^+^, Ca^2+^, Mg^2+^, and Zn^2+^ on T26GAL activity was determined by individual addition of 10 μl of 50 mM KCl, NaCl, CaCl_2_, MgCl_2_, and ZnCl_2_ solutions into 100 μl enzyme solution, respectively. The control reaction performed in 0.01 M sodium phosphate buffer (pH 7.0) in the absence of any of the metal ions was defined as 100% to calculate the relative enzyme activity.

### Determination of T26GAL Substrate Specificity

The 1% (w/v) substrate solutions of locust bean gum, raffinose, guar gum, and stachyose were prepared with 0.01 M sodium phosphate buffer (pH 7.0). Then, 0.1 ml crude enzyme solution and 0.7 ml substrate solution were incubated at 60°C for 30 min. After prompt cooling of the reaction mix, 1.5 ml of DNS was added, and the solution was boiled for 5 min. The solution was adjusted to a total volume of 25 ml with distilled water, and its absorbance was measured at 400 nm. One enzyme unit (U) was defined as the release of 1 μg of galactose per minute.

### Cloning and Heterologous Expression of *T26gal*

The primers (*T26galF* 5′-GGAGATACATATGGGATTATCTATGGTCCAATC-3′ and *T26gal*R 5′-GTGGTGCTCGAG ACGAGCTGCTTTTAACCGC-3′) were used to amplify the coding sequence of α-galactosidase *T26gal* with a PCR Platinum SuperFi Mix purchased from GenStar Biosolutions Co., Ltd. (GenStar, Beijing, China). The PCR thermal cycling program used was as follows: 94°C for 10 min; 30 cycles of 94°C for 30 s, 52°C for 30 s, 72°C for 90 s, and 72°C for 10 min. The plasmids pET-26b (+) were digested with *Nde*I and *Xho*I (Thermo Scientific Corp.). The purified linearized plasmids were ligated using a Seamless Cloning Kit (Thermo Scientific) with the target gene fragment and transformed into competent *E. coli* BL21 (DE). The transformants were selected on LB agar plates containing 50 μg/ml of kanamycin and identified by PCR and restriction enzyme digestion, followed by sequencing. The positive transformants were cultured in LB broth with 50 μg/ml of kanamycin to logarithmic phase (OD_600nm_ = 0.6), and the expression of *T26gal* was induced with 0.1 mM isopropyl β-D-1-thiogalactopyranoside (IPTG, Thermo Scientific) for 7 h and assessed by SDS-PAGE electrophoresis. The IPTG-induced BL21/pET26b(+) cell pellets were re-suspended in 0.01 M sodium phosphate buffer (pH 7.0), and disintegrated using an ultrasonic cell disintegrator (Ningbo Xinzhi Instrument Inc., China). The recombinant α-galactosidase T26GAL with C-terminal His-Tag was purified with Ni-affinity column chromatography [20].

### Bioinformatics Analysis

The coding sequence of *T26gal* has been submitted to NCBI GenBank (Accession No. BankIt2577013 T26-galactosidase ON368188). The physicochemical properties of T26GAL were predicted according to the amino acids sequence using DNAman software [21], and the prediction of signal peptides was performed using SignalP5.0 [20] (https://services.healthtech.dtu.dk/service.phpSignalP-5.0). Pfam [22] (http:// pfam.xfam.org/) and Swiss-Model [23] (https://swissmodel.expasy.org/) were used to predict the protein structure, and amino acid sequences were aligned using NCBI Protein blast [24] (https://www.ncbi.nlm.nih.gov/protein/).

### Statistical Analyses

All the experiments were performed in triplicate. Data were processed using Origin 9.0 software (Origin Lab Corp., USA). A one-way analysis of variance (ANOVA) with the Student-Newman-Keuls method was performed in SPSS, with a *p*-value < 0.05 indicating statistically significant differences.

## Results

### Identification of the Isolate T26

**Fig. 1:**
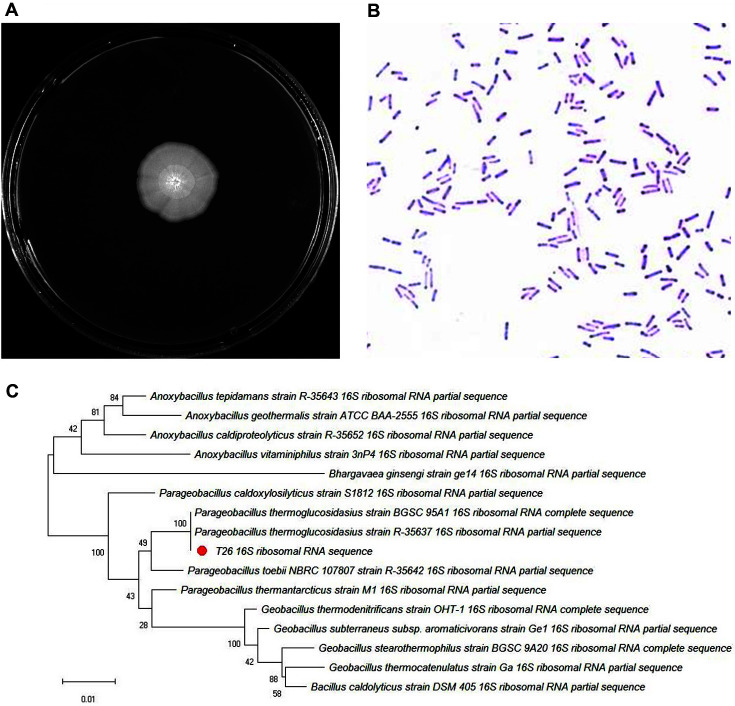
Identification of the strain T26. (**A**) Morphology of the T26 colony; (**B**) Microscopic morphology assessment after Gram staining; (**C**) Phylogenetic tree of T26 strain based on the 16S rDNA sequence.

The T26 isolate from the thermophilic lignocellulolytic microbial consortium TMC7 was identified to form opaque colonies with a rough, flat, and irregular edge (Fig. 1A). Further examination revealed that the T26 strain was a straight, rod-shaped bacterium with terminal endospores. Gram staining of the isolate resulted in bluish purple-colored cells, indicating that it was a gram-positive strain (Fig. 1B). Subsequently, the homologous alignment of the 16S rDNA sequences was performed and a phylogenetic tree was plotted (Fig. 1C). The results of the analyses showed that the T26 isolate was closely related to *P. thermoglucosidasius*.

The biochemical properties of T26 were analyzed using the API test. The ability of strain T26 in fermenting 49 types of carbohydrates was examined and the results, shown in Table 1, revealed that the T26 isolate could ferment various sizes of carbohydrate monomers, *i.e.*, 5C (D-xylose and L-arabinose), 6C (D-galactose, D-glucose, D-fructose, and D-mannose), and methylated glycosides (methyl-β-D-Xylopyranoside and methyl-α-D-glucopyranoside). Moreover, the fermentation also occurred on disaccharides (D-cellobiose, D-maltose, D-melibiose, D-saccharose, D-trehalose, D-turanose, and gentiobiose), trisaccharides (D-melezitose and D-raffinose), and polysaccharides (inositol, starch, and glycogen). Additionally, the T26 isolate could ferment alditols (D-mannitol, D-sorbitol, xylitol) and N-acetylglucosamine, which may be associated with the debranching of hemicellulose. Additionally, the T26 isolate could ferment heteropolysaccharide (inulin) and aromatic glycosides (esculin ferric citrate, amygdalin, arbutin, and salicin), which may be involved in the degradation of lignin-carbohydrate complex. Overall, these results showed that the T26 isolate could utilize almost all the reported substrates of *Geobacillus* species [25, 26]. Interestingly, we found that besides the reported substrates, the T26 isolate exhibits a much wider trophism than other *Geobacillus* species. Indeed, the present study is the first report to demonstrate the fermentation of some substrates by T26, such as methyl-β-D-xylopyranoside, D-sorbitol, methyl-α-D-glucopyranoside, amygdalin, D-melibose, inulin, D-melezotose, D-raffinose, potassium gluconate, and xylitol, among the *Geobacillus* species.

### Growth Characteristics of the T26 Strain

**Fig. 2:**
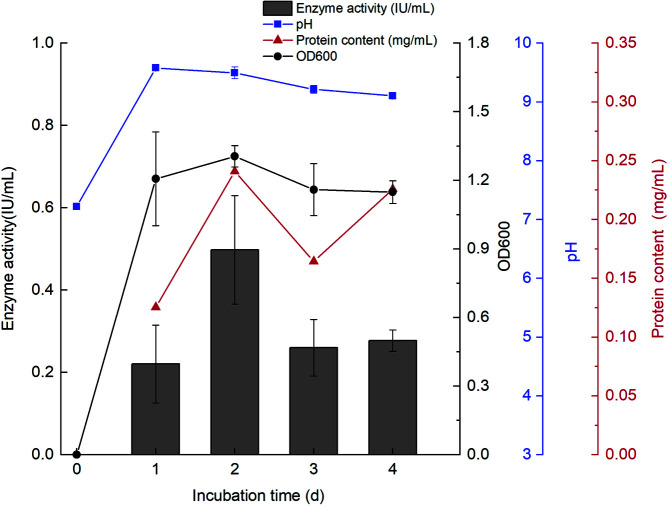
Characteristics of the strain T26. The dynamics of OD_600nm_, pH, supernatant protein content, and supernatant α- galactosidase activity during 1–4 days of T26 culture. The data represent the mean and standard deviation from three independent experiments.

The growth characteristics of the T26 strain were examined using the classical growth curve analysis. The results revealed that during T26 culture, the biomass peaked at 2 days after the log phase, and then declined (Fig. 2). Furthermore, the pH of the culture media was also quickly found to be increased at the beginning of the log phase, reaching the maximum value of 9.58 at day 1, and then gradually decreasing. Interestingly, the T26 culture had always maintained alkaline pH during the whole cultivation. Likewise, the trend of protein concentration generally corroborated with that of the T26 biomass. The highest protein content of 0.24 mg/ml in the culture supernatants was observed at day 2. Additionally, the maximum α-galactosidase activity of 0.50 IU/ml in the supernatants was observed at day 2, with a specific activity of 2.07 IU/mg.

### Analysis of α-Galactosidase T26GAL and Its Coding Gene *T26gal*

**Fig. 3:**
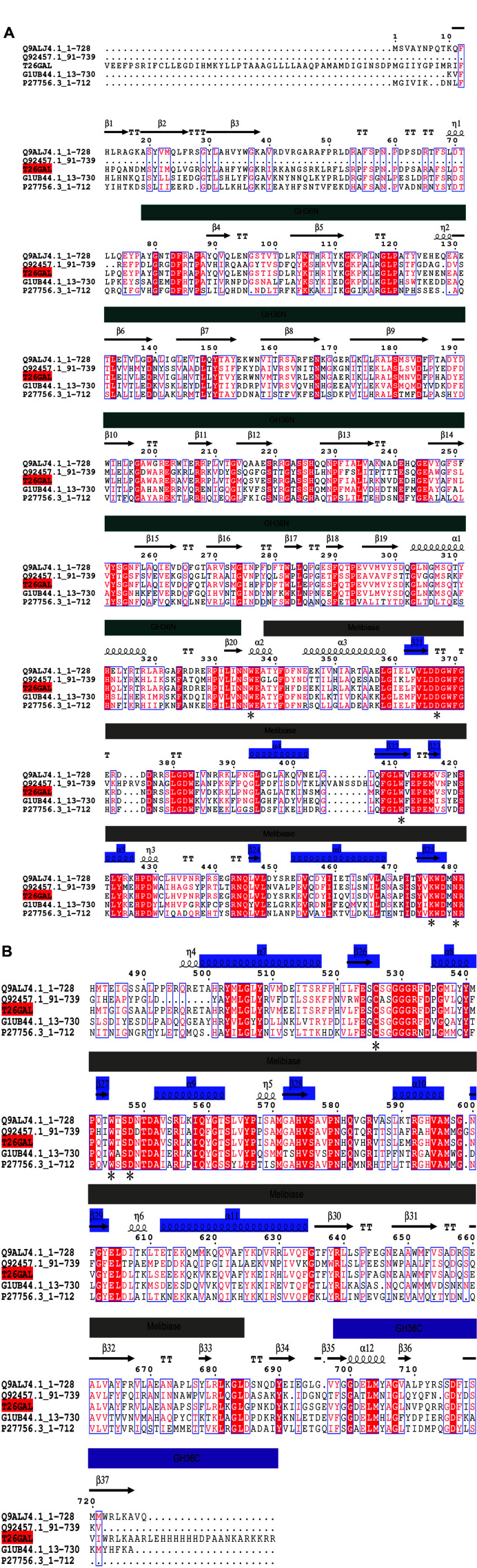
Analysis of α-galactosidase T26GAL. The three structural domains of T26GAL are indicated by bars with different colors: navy for GH36N, grey for melibiase, purple for GH36C; * indicates the WDWKNCWD active site of T26GAL consistent with the conserved domain of GH36 family; the blue bars indicate the eight α/β central barrels; alignment of amino acid sequences of glycoside hydrolases derived from the GH36 family was performed, with Q9ALJ4.1:1-728 derived from *Geobacillus stearothermophilus*, G1UB44.1:13-730 derived from *Lactobacillus acidophilus* NCFM, P27756.3:1-712 derived from *Streptococcus mutans* UA159, Q92457.1:91-739 derived from *Trichoderma reesei*.

The *T26gal* gene has a length of 2,187 bp while T26GAL, the protein it encodes, is composed of 729 amino acids. The theoretical molecular weight of this protein was determined to be 83.47 kDa, and its isoelectric point was identified to be 8.3. Our SignalP analysis had predicted that the T26GAL protein did not possess any signal peptide. Moreover, the protein sequence of T26GAL exhibited 99% similarity with WP_125009601.1 (α-galactosidase from *P. thermoglucosidasius*). Pfam predictions indicated that the protein sequence of T26GAL had three domains, *i.e.*, GH36-N, melibiase, and GH36-C (Fig. 3). In addition, there are eight β/α barrels in the melibiase active center of T26GAL [27]. Compared with the amino acid sequences of other glycoside hydrolases, the T26GAL protein had an active site, WDWKNCWD, which was consistent with the conserved domain of GH36 family (Fig. 3) [28].

### Cloning of *T26gal* and Its Heterologous Expression in *E. coli* BL21 (DE)

**Fig. 4:**
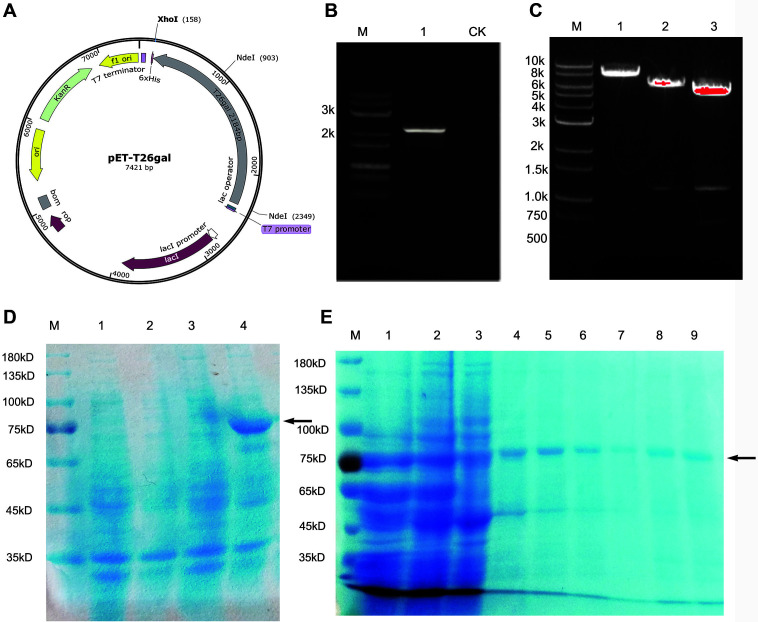
Cloning and heterologous expression of *T26gal*. (**A**) Schematic presentation of the pET-*T26gal* plasmid; (**B**) Cloning of the *T26gal* gene (M: DM0004 DNA Marker; 1: *T26gal* gene; CK: blank control); (**C**) Restriction enzyme digestion verification of pET-*T26gal* (M: DM0005 DNA Marker; 1: Digestion by *Xho*I; 2: Digestion by *Nde*I; 3: Double digestion by *Xho*I/ *Nde*I; (**D**) IPTG-induced expression of T26GAL in *E. coli* BL21 (M: Standard molecular weight of protein 1: Total protein of BL21/pET26b(+) before IPTG induction, 2: Total protein of BL21/pET26b(+) after IPTG induction, 3: Total protein of BL21/ pET-*T26gal* before IPTG induction, 4: Total protein of BL21/pET-*T26gal* after IPTG induction, the arrow indicates the target protein. (**E**) Ni-affinity column chromatography purification of the recombinant α-galactosidase T26GAL (M: Standard molecular weight of protein, 1 and 2: The extraction of IPTG induced BL21/pET26b(+) cell pellets, 3: the sample after passing Ni-affinity column, 4 and 5: the washout of the Ni-affinity column with 100 mM imidazole, 6 and 7: the washout of the Ni-affinity column with 200 mM imidazole, 8 and 9: the washout of the Ni-affinity column with 300 mM imidazole).

The α-galactosidase-encoding gene *T26gal*, with a length of 2,184 bp, was amplified using the primer pair *T26galF*/*T26gal*R with the genomic DNA of T26 serving as a template. The amplified *T26gal* insert was ligated into the *Xho*I/*Nde*I site of the pET-26b (+) vector using a Seamless Cloning Kit and transformed into the competent *E. coli* BL21 (DE) (Figs. 4A and 4B). The plasmid pET-*T26gal* was extracted from the transformants and identified with restriction enzyme analysis, the results of which demonstrated that digestion of pET-*T26gal* with *Xho*I generated a product of 7,421 bp (Fig. 4C, lane 1). Furthermore, due to the presence of a *Nde*I site at 745 bp in *T26gal*, the digestion of pET-*T26gal* with *Nde*I generated two fragments of 1,446 bp and 5,975 bp (Fig. 4C, lane 2). However, double digestion of pET-*T26gal* with *Nde*I and *Xho*I generated three fragments of 745 bp, 1,446 bp, and 5,230 bp (Fig. 4C, lane 3). Thus, the analyses with restriction enzyme digestion confirmed the identification and successful construction of the pET-*T26gal* expression vector. Furthermore, the IPTG-induced expression of *T26gal* was performed and verified by SDS-PAGE electrophoresis (Fig. 4D). Subsequently, the C-terminal His6-Tag from pET-26b (+) facilitated the purification of the recombinant α-galactosidase T26GAL by Ni-affinity column chromatography. Further analysis revealed that the size of the purified protein was consistent with the predicted 83.47 kDa molecular mass of T26GAL (Fig. 4E).

### Enzymatic Properties of α-Galactosidase T26GAL

**Fig. 5:**
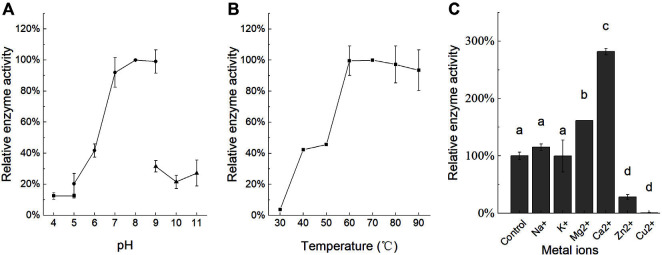
Enzymatic properties of T26GAL. **A**, **B**, and **C** indicate the effect of pH (pH 4.0–5.0 in disodium hydrogen phosphate-citrate buffer, pH 5.0–9.0 in phosphate buffer, pH 9.0–11.0 in sodium hydroxide-glycine buffer), temperature, and metal ions on α-galactosidase enzyme activity, respectively. The data represent the mean and standard deviation from three independent experiments. Values with different letters indicate significant differences (one-way ANOVA with the Student- Newman-Keuls method, *p* < 0.05).

The enzymatic properties of α-galactosidase T26GAL were assayed using the purified recombinant protein. Our studies revealed the optimum pH of T26GAL to be 8.0, and that the relative enzyme activity was greater than 80% at pH 7.0–9.0 (Fig. 5A). In the temperature range of 30–60°C, the enzyme activity of T26GAL demonstrated an increasing trend with the increase in temperature. However, the highest enzyme activity of T26GAL was observed at 60°C. Interestingly, T26GAL also exhibited a remarkable thermostability with more than 93% of the relative enzyme activities at higher temperatures of up to 90°C (Fig. 5B).

Further examination of the effect of metal ions on the T26GAL activity revealed that Na^+^ and K^+^ ions had no significant effect, whereas Mg^2+^ and Ca^2+^ ions significantly promoted the activity of α-galactosidase at a concentration of 5 mM, which increased the enzyme activity to 161.59 and 281.61% of the original activity, respectively. In contrast, the Zn^2+^ and Cu^2+^ ions at a concentration of 5 mM considerably inhibited the enzyme activity of α-galactosidase, reducing it to 28.44 and 0.82% of the original activity, respectively (Fig. 5C).

### Substrate Specificity of α-Galactosidase T26GAL

**Fig. 6:**
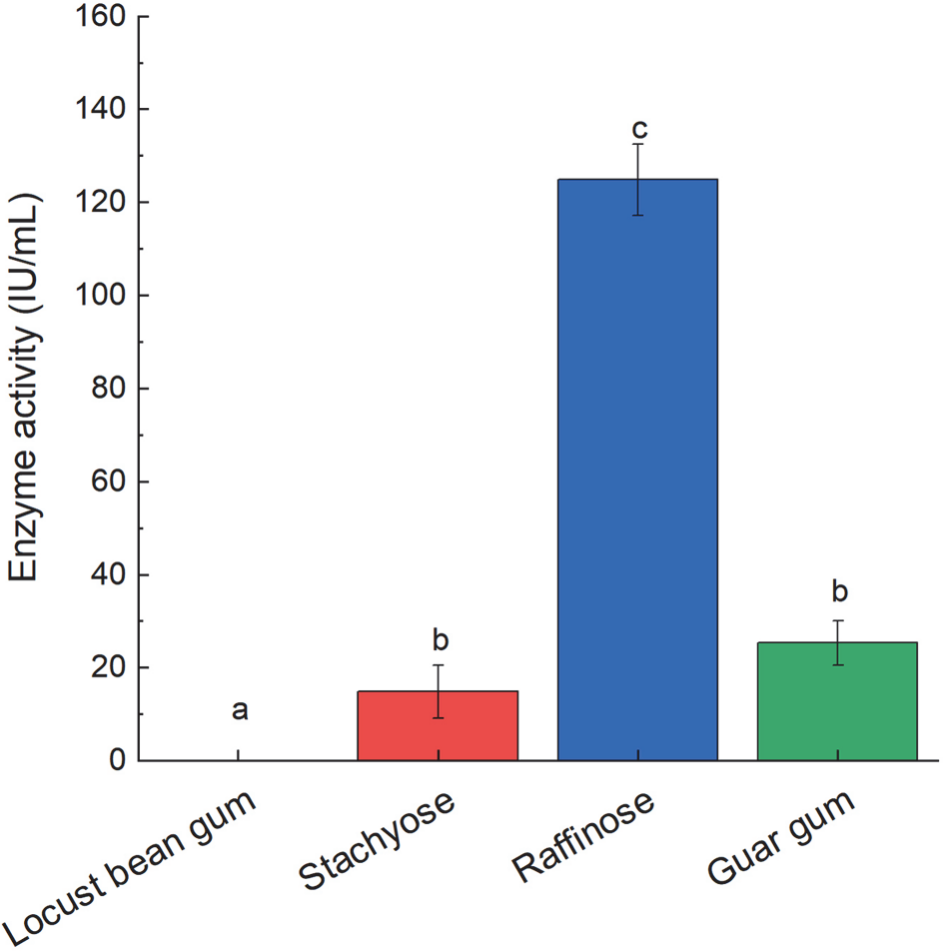
Substrate specificity of α-galactosidase. The data represent the mean and standard deviation from three independent experiments. Values with different letters indicate significant differences (one-way ANOVA with the Student- Newman-Keuls method, *p* < 0.05).

To assess the substrate specificity of T26GAL, the decomposition of locust bean gum, raffinose, guar gum, and stachyose by T26GAL was performed (Fig. 6). The results showed that T26GAL efficiently decomposed the stachyose, raffinose, and guar gum, but not the locust bean gum. Furthermore, the T26GAL decomposition activity toward raffinose was the highest (124.78 IU/ml), followed by guar gum (25.32 IU/ml), and the activity in decomposing stachyose was the lowest (14.83 IU/ml).

## Discussion

According to the CAZy database (www.cazy.org), α-galactosidases mainly belong to GH27 and GH36 among the glycoside hydrolase families. Therein, most of the fungal-derived galactosidases belong to the GH27 family, while most of the bacterial-derived α-galactosidases belong to the GH36 family. In the present study, the theoretical molecular weight of T26GAL was determined to be 83.47 kDa, which is consistent with that of the previously reported GH36 family α-galactosidases, such as the 82.9 kDa α-galactosidase from *Gibberella* sp. F75 [29] and the 81.8 kDa α-galactosidase isolated from *P. janczewskii zaleski* [28]. Homology-modeling using Swiss-Model showed that the 3D structure of T26GAL was similar to the α-galactosidases from *L. acidophilus* and *Thermotoga maritima*. The three domains of T26GAL were an N-terminal β-supersandwich domain (GH36-N) followed by a canonical (β/α)_8_-barrel domain (melibiase) and a C-terminal β-sheet domain (GH36-C) (Fig. S1)[30-32]. Furthermore, four identical monomers of T26GAL were identified to form a tightly packed tetramer through self-association (Fig. S1). Due to the tetrameric assembly, the shallow active site pocket extends toward a deep substrate-binding tunnel formed by the loop regions of the central (β/α)_8_-barrel and loop regions of the N-and C-terminal regions of different subunits. This pocket structure thus provides a platform for efficient substrate binding and confirms the strict specificity of the enzyme for α-1,6-linked galactose [33].

Dey PM. [34] had classified α-galactosidases into groups I and II based on the degree of polymerization of their substrates. The group I α-galactosidases hydrolyzed oligosaccharides such as raffinose and stachyose, whereas the group II α-galactosidases hydrolyzed polysaccharide substrates such as galactomannan and guar gum. The α-galactosidases derived from the GH36 family could hydrolyze synthetic p-nitrophenyl substrates and raffinose family oligosaccharides such as raffinose and stachyose but not the larger polysaccharides [35]. Furthermore, our study has demonstrated that T26GAL is capable of hydrolyzing raffinose and stachyose but not the locust bean gum, a galactose polysaccharide, which was consistent with the properties of the GH36 family of α-galactosidases. Particularly, T26GAL was also able to catabolize the guar gum. The glycosidase catalysis of GH36 α-galactosidases has been documented to adopt a substrate retention mechanism with aspartate or glutamate as cofactors [36]. However, the reason for the division of α-galactosidases into two categories for degrading oligo-oligosaccharides and galactomannans remains unclear. The study of the degradation mechanism by co-crystallization of the substrate, for instance, may help to understand the relationship between the α-galactosidase structure and their mechanism of substrate degradation [37].

For the stringent reaction temperature prerequisite in the industrial application of α-galactosidases, great effort has been made to identify and characterize novel thermophilic α-galactosidases. Huang *et al*. [38] reported that galactosidase AgaB with an optimum temperature of 37°C exhibited no activity over 60°C. The optimum temperature of the galactosidase LrAgal36A cloned from the *Lichtherimia ramosa* was 65°C [7], while the same demonstrated less than 10% of the relative enzyme activity at 80°C. The optimum reaction temperature of both galactosidase rILgalA [39] and rCbAga36 [40] has been reported to be 70°C, with a drastic decrease in the enzyme activity at temperatures below 60°C and above 75°C. Compared with other α-galactosidases in the GH36 family, T26GAL shows a wide range of optimum temperatures. The optimum temperature of the galactosidase T26GAL in the present study was determined to be 60°C, and more than 90% of enzyme activity was retained at temperatures ranging from 60–90°C. The remarkable thermostability endowed T26GAL with a special advantage for potential application in high-temperature scenarios such as sugar refining, soya processing, feed supplementation, and guar gum processing [15, 41].

According to the 16S rDNA sequencing analysis, the T26 isolate was identified as *P. thermoglucosidasius*, which was once categorized under the genus *Geobacillus* and subsequently renamed as a new species called *P. thermoglucosidasius* in 2016 by Aliyu *et al*. [42] using AAI (average amino acid identity), ANI (average nucleotide identity), and dDDH (digital DNA-DNA hybridization). This finding indicates that in the present study, the T26 isolate corresponded to the dominant genus *Geobacillus* in the microbial consortium TMC7 as identified by metagenomic analysis in our previous study [5]. Previous metagenomic analysis has shown that *Geobacillus* encoded enzymes are associated with the debranching of hemicellulose. Since hemicellulose contains heteropolysaccharides with different side chains, the catabolism of hemicellulose requires various debranching enzymes [43]. Significant synergistic effects between α-galactosidases and mannosidase have been documented in the degradation of carob gum [44]. The α-galactosidase is known to cleave the α-galactose attached to the mannan backbone, allowing mannosidase to be more accessible to the mannan backbone for its hydrolytic actions. Furthermore, the soluble sugar released induces the highly efficient enzyme activity, thereby promoting the degradation of the backbone. In this study, the API tests showed that T26 could ferment a variety of substrates (Table 1), suggesting that this strain may serve as a generalist during the degradation of lignocellulose by the microbial consortium TMC7. We speculated that it might be caused by the evolution process during the construction of TMC7. Furthermore, bacteria are known to acquire novel DNA through the process of horizontal gene transfer (HGT), providing a competitive edge against other organisms within the microbial community [45]. As a surviving victor in the TMC7 consortium, the generalist function of the T26 isolate might be attributable to its survival strategy.

In conclusion, the present study demonstrated the isolation of *P. thermoglucosidasius* from the lignocellulolytic microbial consortium TMC7. Subsequently, the heterologous expression of α-galactosidase T26GAL was performed along with biochemical characterization. Our results revealed that T26GAL is a thermophilic enzyme that can degrade raffinose, stachyose, and guar gum and is well adapted to a wide temperature and pH range. Especially, the remarkable thermostability endows T26GAL with a special advantage for its potential industrial application. Moreover, we showed that the T26 isolate could ferment a variety of carbohydrates, which may be attributed to the degradation of cellulose, hemicellulose and lignin-carbohydrate complex. Overall, the isolation of the T26 strain and the α-galactosidase T26GAL achieved in this study provides an experimental foundation for subsequent studies delineating the synergy between the lignocellulolytic enzyme complexes present in the microbial consortium TMC7.

## Supplemental Materials

Supplementary data for this paper are available on-line only at http://jmb.or.kr.

## Figures and Tables

**Table 1:** Biochemical characterization of T26 and differentiation from other *Geobacillus* species.

Characteristic	1	2	3	4	5	6	7
Glycerol	-	w	w	-	-	+	+
Erythritol	-	-	-	nd	nd	nd	nd
D-arabinose	-	-	-	nd	nd	nd	nd
L-arabinose	+	-	w	-	-	+	-
D-ribose	-	w	w	nd	nd	+	+
D-xylose	+	-	w	v	+	-	-
xylose	-	-	-	nd	nd	nd	nd
Adonite	-	-	-	v	+	-	-
Methyl-β-D-Xylopyranoside	+	-	-	nd	nd	nd	nd
D-galactose	+	w	w	+	v	+	+
D-glucose	+	+	+	+	+	nd	nd
D-fructose	+	+	+	+	+	nd	nd
D-mannose	+	+	+	nd	nd	nd	nd
L-sorbose	-	-	-	nd	nd	nd	nd
L-rhamnose	-	-	w	-	-	-	-
Dulcitol	-	-	-	nd	nd	nd	nd
Inositol	+	-	-	-	+	-	-
D-mannitol	+	-	w	+	+	+	+
D-sorbitol	+	-	w	-	-	-	-
Methyl-α-D-mannopyranoside	-	-	-	nd	nd	nd	nd
Methyl-α-D-glucopyranoside	+	w	w	nd	nd	-	-
N-acetylglucosamine	+	-	+	nd	nd	nd	nd
Amygdalin	+	-	w	nd	nd	-	-
Arbutin	+	-	+	nd	nd	-	-
Esculin ferric citrate	+	w	+	nd	nd	nd	nd
Salicin	+	w	+	nd	nd	-	+
D-cellobiose	+	w	w	+	+	+	+
D-maltose	+	+	+	nd	nd	nd	nd
D-lactose	-	-	-	-	-	-	-
D-melibiose	+	w	-	nd	nd	-	-
D-saccharose	+	+	w	nd	nd	nd	nd
D-trehalose	+	w	+	nd	nd	+	+
Inulin	+	-	-	nd	nd	nd	nd
D-melezitose	+	w	-	nd	nd	nd	nd
D-raffinose	+	w	-	nd	nd	-	-
Starch	+	w	w	+	+	nd	nd
Glycogen	+	-	-	nd	nd	-	+
Xylitol	+	-	-	nd	nd	nd	nd
Gentiobiose	+	w	w	nd	nd	-	-
D-turanose	+	w	w	nd	nd	+	-
D-lycose	-	-	-	nd	nd	nd	nd
D-tagatose	-	-	-	nd	nd	nd	nd
D-fucose	-	-	-	nd	nd	nd	nd
L-fucose	-	-	-	nd	nd	nd	nd
D-arabitol	-	-	-	nd	nd	nd	nd
L-arabitol	-	-	-	nd	nd	nd	nd
Potassium gluconate	+	-	-	nd	nd	nd	nd
Potassium 2-ketogluconate	-	-	-	nd	nd	nd	nd
Potassium 5-ketogluconate	-	-	-	nd	nd	nd	nd

Strains: 1, T26; 2, *Geobacillus stearothemophilus*; 3, *G. thermoglucosidasius*; 4, *G. thermoleovorans*; 5, *G. kaustophilus*; 6, *G. jurassicus*; 7, *G. subterraneus*. Data for T26 were obtained in the present study. Data for 2 and 3 were taken from the API database; for 4 from Ulya *et al*. [25]; for 5–7 from Semenova, *et al*. [26]. +, Positive; -, Negative; w, Weakly positive; v, Variable within the group; nd, No available data.
