# Supplementary material for: Heterologous Expression of a Thermostable α-Galactosidase from Parageobacillus thermoglucosidasius Isolated from the Lignocellulolytic Microbial Consortium TMC7
Source: J Microbiol Biotechnol. 2022 May 16;32(6):749–60. doi: 10.4014/jmb.2201.01022 (PMC9628905; doi:10.4014/jmb.2201.01022)
Supplement: Supplementary file 1 [file jmb-32-6-749-supple.pdf]

J. Microbiol. Biotechnol.
https://doi.org/10.4014/jmb.2201.01022

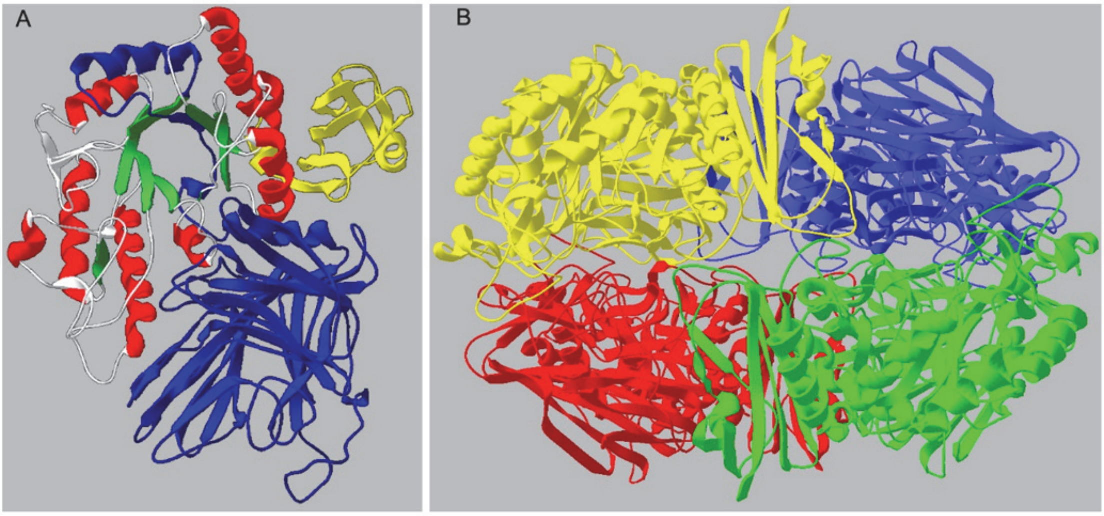

Supplementary Figures S1. The predicted structure of T26GAL. (A) The three domains of T26GAL monomer: N-terminal β-supersandwich domain GH36-N marked in blue, canonical $(\beta/\alpha)_8$-barrel domain marked in red/green, and C-terminal β-sheet domain GH36-C marked in yellow; (B) The tetramer packed by four identical monomers.
